# Potentiating the effects of radiotherapy in rectal cancer: the role of aspirin, statins and metformin as adjuncts to therapy

**DOI:** 10.1038/bjc.2017.175

**Published:** 2017-06-22

**Authors:** K J Gash, A C Chambers, D E Cotton, A C Williams, M G Thomas

**Affiliations:** 1 School of Cellular and Molecular Medicine, University of Bristol, Bristol BS1 8TD, UK; 2 Department of Coloproctology, University Hospitals Bristol NHS Foundation Trust, Bristol BS2 8HW, UK

**Keywords:** colorectal cancer, radiotherapy, adjunct, tumour regression, therapy response, aspirin, statins, metformin

## Abstract

**Background:**

Complete tumour response (pCR) to neo-adjuvant chemo-radiotherapy for rectal cancer is associated with a reduction in local recurrence and improved disease-free and overall survival, but is achieved in only 20–30% of patients. Drug repurposing for anti-cancer treatments is gaining momentum, but the potential of such drugs as adjuncts, to increase tumour response to chemo-radiotherapy in rectal cancer, is only just beginning to be recognised.

**Methods:**

A systematic literature search was conducted and all studies investigating the use of drugs to enhance response to neo-adjuvant radiation in rectal cancer were included. 2137 studies were identified and following review 12 studies were extracted for full text review, 9 studies were included in the final analysis.

**Results:**

The use of statins or aspirin during neo-adjuvant therapy was associated with a significantly higher rate of tumour downstaging. Statins were identified as a significant predictor of pCR and aspirin users had a greater 5-year progression-free survival and overall survival. Metformin use was associated with a significantly higher overall and disease-free survival, in a subset of diabetic patients.

**Conclusions:**

Aspirin, metformin and statins are associated with increased downstaging of rectal tumours and thus may have a role as adjuncts to neoadjuvant treatment, highlighting a clear need for prospective randomised controlled trials to determine their true impact on tumour response and overall survival.

Advances in surgical resection (MacFarlane *et al*, 1993; Holm *et al*, 2007), development of adjuvant therapies (Gray *et al*, 2007; Sebag-Montefiore *et al*, 2009; Yaffee *et al*, 2015) and efforts to optimise early detection (National Cancer Intelligence Network, 2009), have led to a reduction in rectal cancer mortality rate (Office for National Statistics, 2014). Despite this, there are still high rates of local recurrence and systemic disease, which contribute to 5-year survival rates of less than 60% (Office for National Statistics, 2014). Poor response to adjuvant and neo-adjuvant treatment affects around 30–40% of patients and continues to pose a significant problem. Drug repurposing for anti-cancer treatments is gaining momentum, yet research into the utilisation of drugs as adjuncts, to enhance response to chemo/radiotherapy, is more limited. The purpose of this review is to explore evidence in the literature for such adjuncts; drugs that have been shown to increase tumour regression by enhancing the response of neo-adjuvant therapy in the management of patients with rectal cancer.

Concerns regarding the high rates of local recurrence of rectal cancer (Heald *et al*, 1982; Pilipshen *et al*, 1984; Nymann *et al*, 1995; den Dulk *et al*, 2009; Shihab *et al*, 2010) have catalysed the evolution of operative techniques, to facilitate more ‘radical rectal resections’, (e.g., Total Mesorectal Excision (TME) (MacFarlane *et al*, 1993) and Extra-Levator Abdomino-Perineal Excision (ELAPE) (Holm *et al*, 2007)). In addition, studies have examined the use of adjuvant and neo-adjuvant chemo- and/or radio-therapy, to ascertain which modality or combinations confer optimum benefit. Currently, in the UK, patients with rectal tumours at high risk of local recurrence (i.e., threatened mesorectal resection margins or lymph node involvement, Table 1) are offered neo-adjuvant long-course chemo-radiotherapy (LCRT), followed by an interval to surgery to maximise response. The aims are to reduce tumour bulk to facilitate resection with a clear circumferential resection margin (CRM), minimise disease recurrence, and to increase sphincter-preserving surgery (NICE, 2014). Those deemed to be moderate risk (Table 1) are offered short-course preoperative radiotherapy (SCPRT) immediately prior to surgery. Numerous studies have now demonstrated that neo-adjuvant treatment improves oncological outcomes (Pahlman *et al*, 1997; Kapiteijn *et al*, 2001; Rullier *et al*, 2001; Sauer *et al*, 2004; Bosset *et al*, 2006; Peeters *et al*, 2007).

Tumour regression in response to neo-adjuvant chemo-radiation (Mandard *et al*, 1994; Wheeler *et al*, 2002) varies significantly between patients; some tumours undergo complete regression while others demonstrate no response (Dhadda *et al*, 2011). This can be graded by various systems, but commonly used is the American Joint Committee on Cancer (AJCC) criteria (Table 2), with regression classified as 0–3, where 0 equates to a pathological complete response (pCR). The reasons for the disparity between patients in tumour regression are poorly understood. Several studies have investigated predictors of response, but have only demonstrated minor associations and reported inconsistent findings (de Campos-Lobato *et al*, 2011; Martin *et al*, 2012).

Around 20–30% of tumours have a pCR (Mace *et al*, 2015), which is associated with a reduction in local recurrence and improved disease-free and overall survival (Rödel *et al*, 2005; Roh *et al*, 2009; Habr-Gama *et al*, 2010, 2013). Although pCR can be dependent on the quality of the surgical specimen and the accuracy of pathological examination can vary between pathologists (Chua, 2010), there appears to be increasing evidence to suggest that patients with pCR could be safely managed by local excision. Local procedures, such as Transanal Endoscopic Microsurgery (TEMS) or Transanal Endoscopic Operation (TEO), or indeed surveillance (‘watch and wait’ strategy), can potentially avoid the morbidity associated with resectional surgery and enable organ preservation (Han *et al*, 2015; Ryan *et al*, 2016). A recent systematic review suggests that local excision after neoadjuvant therapy for rectal cancer should only be considered as curative if complete pathological response is obtained, as pooled local recurrence rates were significantly lower and median disease-free survival significantly higher, for ypT0 tumours compared with ypT1 tumours or higher (Bach *et al*, 2009).

Therefore, optimising response to radiation is now fundamental to improving outcomes from rectal cancer, in both early and locally advanced stages. Studies are currently underway to extend the role of neo-adjuvant radiotherapy prior to the local excision of early rectal tumours, with the aim of treating microscopic lymph node metastases and reducing local recurrence (Leong *et al*, 2011, 2014). To identify patients whose tumours may be appropriate for local excision, studies have investigated the utilisation of biomarkers to predict mesorectal lymph node metastases. These studies have identified several biomarkers that have early promise, including the methylation of retinoic acid receptor *β* (RAR*β*), chemokine ligand 12 (CXCL12), and death-associated protein kinase 1 (DAPK1) (TREC Trial Protocol, 2010; Leong *et al*, 2011, 2014). Potentially, radiation followed by local excision for early rectal cancers in patients with confirmed lymph node negativity may avoid resectional surgery in this patient group. This concept is currently being further explored in the TREC and future STAR-TREC trial (STAR-TREC Trial Protocol, 2016).

As malignancies develop they acquire both epigenetic and genetic changes that lead to therapy resistance and may promote evasion of apoptosis, an important ‘hallmark of cancer’ (Hanahan and Weinberg, 2011). The role of ‘targeted adjuncts’ to increase therapeutic response is becoming increasingly recognised. This concept suggests that established drugs with known anti-cancer properties, (but which are not primarily chemo-therapeutic agents), could be administered during neo-adjuvant therapy to sensitise cells and thus augment tumour response.

## Materials and methods

**Figure 1:**
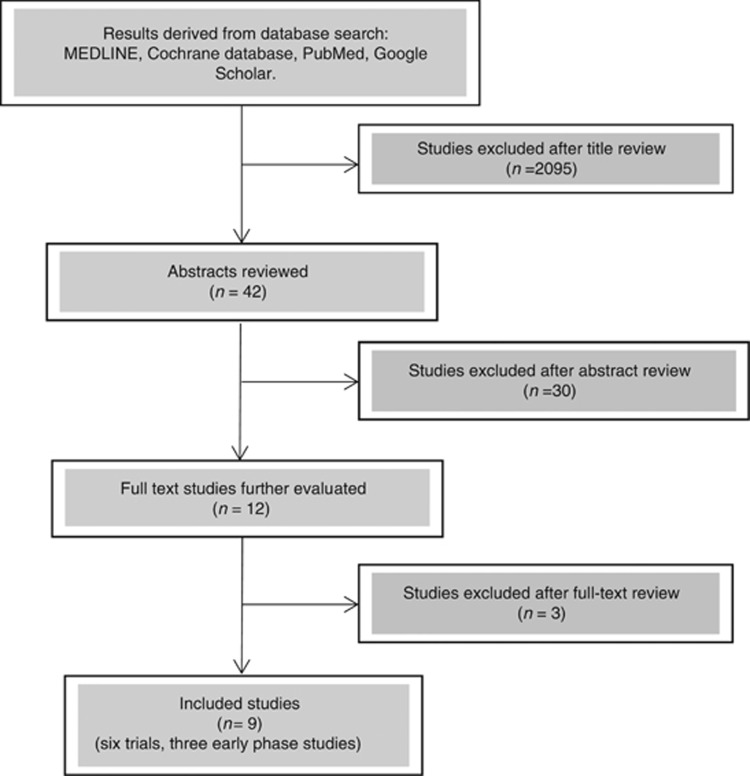
**A PRISMA flow diagram showing the inclusion and exclusion of relevant studies.** Due to the small number of studies yielded by the search criteria, the heterogeneity of outcomes and the diversity of adjuncts assessed, meta-analysis was not possible.

This review examines the literature for potential adjuncts or supplementary treatments that may enhance the pathological response to neo-adjuvant therapy in rectal cancer. Systematic review and meta-analysis was attempted; a search was performed using Medline, Cochrane, Pubmed and Google Scholar databases with secondary searching of identified papers. The search terms used were: rectal cancer, radiotherapy, neoadjuvant, chemoradiation, metformin, aspirin and statins. We identified 2137 papers of which 9 studies were included in the final review (see Figure 1 for PRISMA flow diagram (Moher *et al*, 2009)). Due to the limited number of randomised controlled trials and the heterogeneity of relevant trials a narrative review has been conducted.

## Discussion

### Statins as an adjunct to therapy

**Figure 2:**
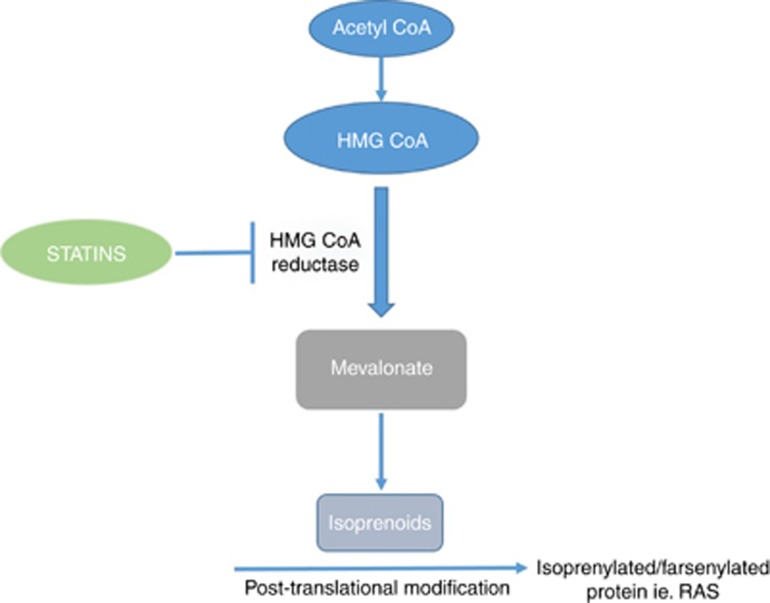
**The mechanism of action of statins on the mevalonate pathway.** Statins are competitive inhibitors of the enzyme 3-hydroxy-3-methyl-glutaryl-coenzyme A reductase (HMG CoA reductase). This is a critical enzyme in the production of mevalonate and inhibition consequently reduces its derivatives, cholesterol, steroids and isoprenoids. Importantly in cancer, inhibition of this pathway reduces specific prenylated proteins, such as RAS.

There is increasing evidence that statins may play an important role in reducing colorectal tumourigenesis, both in terms of disease prevention and improved survival (Siddiqui *et al*, 2009; Bardou *et al*, 2010). The term statin denotes a class of drug that inhibits the mevalonate pathway, which is critical for the production of the majority of organic compounds present in mammalian cells including steroids and cholesterol amongst other isoprenoid derivatives (Goldstein and Brown, 1990). Statins are synthetic inhibitors of hydroxymethylglutaryl coenzyme A reductase (HMGCoAR), a rate-limiting enzyme of the mevalonate pathway (Figure 2). Downstream products in the mevalonate pathway, such as farnesyl pyrophosphate, are molecules called isoprenoids. Isoprenylation (attachment of a molecule such as farnesyl pyrophosphate) is an important post-translational modification of proteins that enables their membrane attachment as part of their normal activity.

Statins were initially thought to drive colorectal carcinogenesis as early epidemiological studies showed an association between low cholesterol levels and increased colorectal cancer (Oliver, 1991). However numerous *in vitro* studies have highlighted that statins have a variety of effects in different tumour types (reviewed by Osmak, (2012)). It is now recognised that dysregulation of the mevalonate pathway itself can drive tumourigenesis in a cholesterol-independent manner (Clendening *et al*, 2010). In colorectal cancer, a number of mechanisms have been suggested for statins’ protective effects. G-proteins such as RAS, important in colorectal cancer (Cox *et al*, 2014), undergo post-translational prenylation and early work suggested that RAS activation could be blocked by targeting this pathway (Schafer *et al*, 1989). Inhibition of the upper mevalonate pathway with lovastatin results in increased apoptosis in colorectal cancer cell lines, which could be abrogated with isoprenoid add-back (Agarwal *et al*, 1999). Moreover, mevastatin is thought to enhance the anti-proliferative effects of butyrate (a dietary short-chain fatty acid) in Caco2 colorectal cancer cells through inducing a cell cycle arrest (Wachtershauser, 2001). In addition to this, colorectal cancer cell invasion and metastasis are also thought to be targeted by statins (Kusama *et al*, 2003; Al-Haidari *et al*, 2014). Importantly in the context of this review, simvastatin has been shown to enhance the effect of radiation in-vitro on colorectal cancer cells by upregulating apoptosis in these cells (Lim *et al*, 2015).

Despite this *in vitro* evidence, it has been difficult to translate this to the bedside. Epidemiological work from the United Kingdom, analysed at two time points, showed that there was no difference in colorectal cancer risk with statin use (Vinogradova *et al*, 2007, 2011). Use of simvastatin as an adjunct to treatment in patients with metastatic colorectal cancer also failed to show any benefit to progression free survival in these patients (Lim *et al*, 2015). Furthermore, targeting KRAS expression, in patients who had KRAS mutant tumours, failed to enhance tumour sensitivity to panitumumab, suggesting that statins could not be used to modulate drug sensitivity in tumours with mutant KRAS (Baas *et al*, 2015).

Clinically, it may be that the beneficial effects of statins can be predicted by single-nucleotide polymorphisms (SNPs), such as the rs12654264 genotype. This genotype has been shown to modify the chemo-preventative effects of statins (Lipkin *et al*, 2010) and failure to control experimentally for this polymorphism could explain the current inconsistent results seen with adjunctive statin use.

Currently, translation of this pre-clinical work has been carried out in two studies analysing response of neo-adjuvant chemo-radiotherapy (Katz *et al*, 2005; Mace *et al*, 2013) and a third paper which explored factors predictive of a pCR and identified statins as a significant predictor (Armstrong *et al*, 2015).

(Katz *et al*, 2005) analysed 349 patients from a prospective institutional database, of whom 33 were taking statins (33/349=9.5%). Overall, 18.7% had a pCR. Univariate analysis showed that there was a non-significant trend towards a higher rate of pCR for statin users (30%) compared with non-users (17%, *P*=0.1). Only lower T-stage was found to be significantly associated with increasing pCR rate. An interesting subgroup analysis showed that of those patients taking statins, concomitant aspirin use was associated with a lower rate of pCR than for statin use alone (8 *vs* 47%). On multivariate regression analysis, having adjusted for concomitant aspirin/NSAID usage, statin use alone was significantly associated with pCR (OR 4.2; 1.7–12.1, *P*<0.003) compared to non-statin use.

Following on from this, Mace *et al* (2013) performed a retrospective cohort study, using a prospectively maintained database of patients with rectal cancer undergoing neo-adjuvant chemo-radiotherapy. They identified 407 eligible patients of whom 99 were statin users (24.3%) and showed that statin users were less likely to exhibit no response to neo-adjuvant therapy (i.e., AJCC Regression Grade 3) (11.1%) compared to non-users (19.8%, *P*=0.049). In both univariate and multivariate analyses, statin use was a significant predictor of AJCC response (Grade 0–1), (OR, 2.25; 95% CI, 1.33–3.82), as was the absence of lymphovascular invasion. Moreover, statin users were more likely to have had a near-complete or complete response (i.e., Regression Grade 0 or 1), (65.7 *vs* 48.7%, *P*=0.004). Despite this, there was no significant difference in pCR rates (AJCC grade 0), between statin users (25.2%) and non-users (20.8%, *P*=0.35). Potential bias in this study was introduced as the statin group were significantly older, had higher BMIs, worse American Society of Anesthesiologists (ASA) grades and a greater proportion of male patients than the non-statin group. Furthermore, there was a higher incidence of lymphovascular invasion in tumours of the statin users (11.1 *vs* 5.2%, *P*=0.04). However, the generalisability of the study benefitted as a variety of different statins were used, including Atorvastatin, Simvastatin, Rosuvastatin, Lovastatin and Pravastatin, during its course.

Finally, a study by Armstrong *et al* (2015) analysed factors predictive of pCR following neo-adjuvant chemo-radiotherapy for rectal cancer. Of 885 patients, 18% of patients had a pCR and on univariate analysis, only lower CEA and higher Hb were associated with a pCR. In addition, multivariate analysis showed statin use was significantly predictive of pCR (OR, 1.72; 95% CI 1.02–2.92, *P*=0.04). Other factors included decreased distance from the anal verge and lower pre-operative CEA. pCR was significantly associated an improved 3-year disease-free survival (HR, 0.31; 95% CI 0.20–0.48, *P*<0.0001) and with overall survival (HR, 0.29; 95% CI 0.17–0.51, *P*<0.0001), compared with non-pCR.

### Aspirin: teaching an old drug new tricks

For over three decades the role of aspirin in reducing colorectal cancer risk has been explored in a number of settings; in particular, for high-risk patients with FAP (Waddell and Loughry, 1983; Giardiello *et al*, 1993), HNPCC (Burn *et al*, 2011), patients already treated for colorectal adenomas (Baron *et al*, 2003), and for colorectal carcinomas (Rothwell *et al*, 2012). Similarly, long-term vascular studies of regular low-dose aspirin users have highlighted the significant reduction in colorectal cancer rates and mortality (Rothwell *et al*, 2010). However, until recently, the use of aspirin as an adjunct to treatment has been somewhat overlooked (Restivo *et al*, 2015).

**Figure 3:**
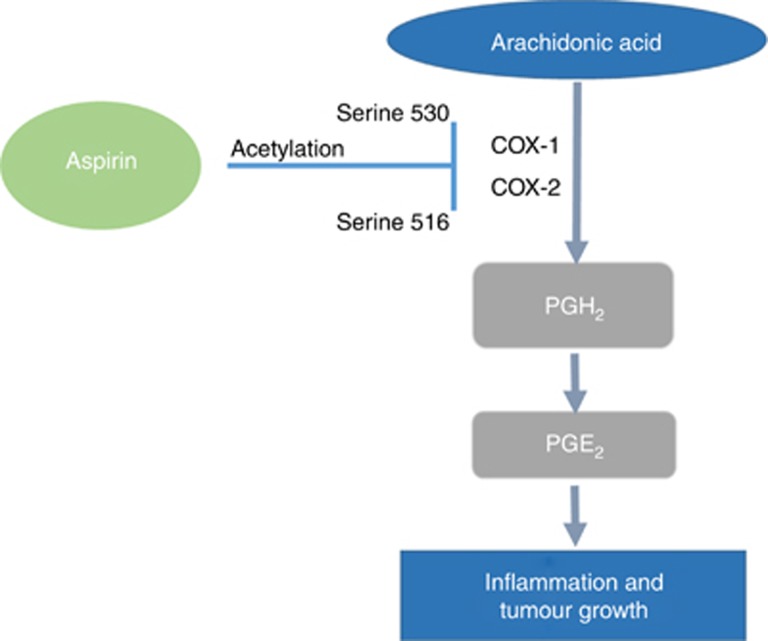
**The cyclo-oxygenase dependent mechanism of action of aspirin.** Aspirin treatment causes the post-translational modification of cyclo-oxygenase (Cox) proteins by acetylating serine residues (Cox-1^Ser530^ and Cox-2^Ser516^) on either Cox-1 & Cox-2 enzymes. This prevents arachidonic acid from accessing the catalytic site on the Cox enzymes. As a result there is reduced formation of the precursor for all prostaglandins (PGs) and therefore lower levels of prostaglandin E_2_ (PGE_2_).

Aspirin inhibits the cyclo-oxygenase (COX) enzymes by the acetylation of serine 530 (COX-1) and of serine 516 (COX-2; Figure 3). This precludes arachidonic acid from accessing the catalytic site on the COX enzyme, preventing it from being metabolised and thus blocking prostaglandin formation (Roth *et al*, 1975). The COX-2-derived prostaglandin, PGE2, is over expressed in colorectal cancer and has been shown to promote tumour growth by binding to the EP transmembrane G-protein-coupled receptors, which activates pathways for gene transcription, proliferation, angiogenesis and inhibits apoptosis (Wang, 2006). This ultimately activates downstream signalling pathways that can increase tumour cell survival, proliferation and migration (Greenhough *et al*, 2009; Wang and DuBois, 2009). Those patients with colorectal cancers that have an increased COX-2 expression, have been found to have reduced 5-year survival, increased metastases and higher rates of local recurrence compared to those without elevated COX-2. Aspirin is thought to potentially exhibit its chemo-preventative properties independently of PGE_2_ through the COX derived antiplatelet effect. Aspirin has been found to be 100 times more powerful at inhibiting platelet COX-1 compared with COX-2 (Piazuelo and Lanas, 2015). Blocking COX-1 activity suppresses thromboxane A_2_ (TXA_2_) synthesis, inhibiting platelet aggregation. Platelets have a limited capacity of protein synthesis, whereas nucleated cells such as monocytes (with COX-2) re-synthesise the enzyme rapidly; enzymes are only temporarily and partially inhibited. Furthermore, aspirin’s half-life is ∼20 min, therefore, only a low dose of around 75 mg is required to maximally inhibit platelets and the effect lasts 24 h, whereas COX-2 suppression necessitates higher and more regular doses.

The cancer stem cell hypothesis suggests that within tumours a small population of cells, colorectal cancer stem cells (CSC), are the driving force behind carcinogenesis: triggering gene transcription; maintaining tumour growth and ultimately fuelling resistance to chemo-radiation and instigating disease recurrence (Cho and Clarke, 2008). The COX-2/PGE_2_ pathway has been shown to stimulate the function and survival of colorectal CSC through up-regulating leucine-rich repeat-containing G-protein-coupled receptor 5 (Lgr5) expression (Al-Kharusi *et al*, 2013). Lgr5 is an intestinal stem cell marker (Barker *et al*, 2007) and a Wnt target gene (Van der Flier *et al*, 2007). Wnt signalling is an essential driving force of the overall biology of the intestinal crypt (Korinek *et al*, 1998). The activation of aberrant Wnt/*β*-catenin signalling is a critical early step in the majority of colorectal cancers with most tumours harbouring APC or *β* -catenin mutations; leading to enrichment of colorectal cancer stem cells (Morin *et al*, 1997).

Tumourigenesis can trigger the production of inflammatory mediators, which result in an increase in prostaglandin synthesis and contribute to the development of an inflammatory microenvironment (Mantovani *et al*, 2008). NF-*κ*B is a key transcription factor in the development of such an environment. NF-*κ*B also has a crucial role in epithelial cells, regulating genes that control cell survival, viral replication and autoimmune function (Bharti and Aggarwal, 2002), thus providing a key link between inflammation and cancer (Karin and Greten, 2005). The release of cytokines and inflammatory signals stimulates the action of NF-*κ*B in epithelial cells, which subsequently promotes the survival of cancer cells. Furthermore, expression of the NF-*κ*B co-regulators, such as BCL-3, has been shown to worsen prognosis in colorectal cancer (Puvvada *et al*, 2010; Saamarthy *et al*, 2015), highlighting the importance of the whole pathway.

To summarise, *in vitro* and *in vivo* pre-clinical work has shown that aspirin may well target a number of different pathways within the tumour cell including the promotion of a cancer stem cell phenotype and the inhibition of tumour microenvironment associated inflammation.

Despite the quantity of pre-clinical studies there are very few studies examining aspirin as an adjunct in patients. Restivo *et al* performed a prospective observational cohort study of all patients with Stage II or III rectal cancer undergoing neo-adjuvant chemo-radiation (Restivo *et al*, 2015). Of the 241 patients identified, 37 in the ‘aspirin-use’ group were compared with 204 controls (no aspirin-use). All 37 patients were taking 100 mg of aspirin as cardiovascular risk prevention, with a median duration of use preoperatively of 5 years. Patients were treated with 45 Gy of radiotherapy over five weeks (as 25 fractions of 1.8 Gy using a 3-field technique with tumour boost of 9 Gy. Chemotherapy was Capecitabine 1650 mg m^−2^. The primary outcome of the study was a ‘good pathological response’ (GPR), defined as downstaging to T0 or T1, N0 disease. GPR was significantly higher in the aspirin group compared to the non-aspirin users (46 *vs* 19%, *P*<0.001). Furthermore, aspirin use was associated with a significantly higher rate of tumour downstaging (to any stage) (67.6 *vs* 43.6%, *P*=0.011). The rate of complete pathological response (pCR) was also higher in the aspirin group, although this did not reach statistical significance (22 *vs* 13%, *P*=0.196). It should be noted that patients in the non-aspirin group had a much higher rate of diagnosis of metastasis pre-operatively. Although the aspirin-use group had a higher median age (71 *vs* 64 years, *P*<0.001) and higher rate of comorbidity (Charlson Score 3–4), (40.5 v 17.6%, *P*<0.004), the two groups were comparable in terms of pre-operative T and N-stage, tumour size, grade and CEA. Aspirin-use was also associated with a better 5-year progression-free survival (86.6 *vs* 67.1% HR=0.20; 95% CI=0.07–0.6) and overall survival (90.6 *vs* 73.2% HR=0.21, 95% CI=0.05–0.89). Cox regression analysis confirmed that aspirin was the only significant factor predictive of progression-free and overall survival.

Interestingly, more patients on aspirin underwent the minimally invasive transanal endoscopic microsurgery (TEM) as their definitive primary surgical procedure (13.5 *vs* 2.5%, *P*=0.017). The authors state that the decision to proceed to TEM was based on significant downstaging by neoadjuvant chemo-radiotherapy.

### Metformin: from folk remedy to anti-cancer drug

**Figure 4:**
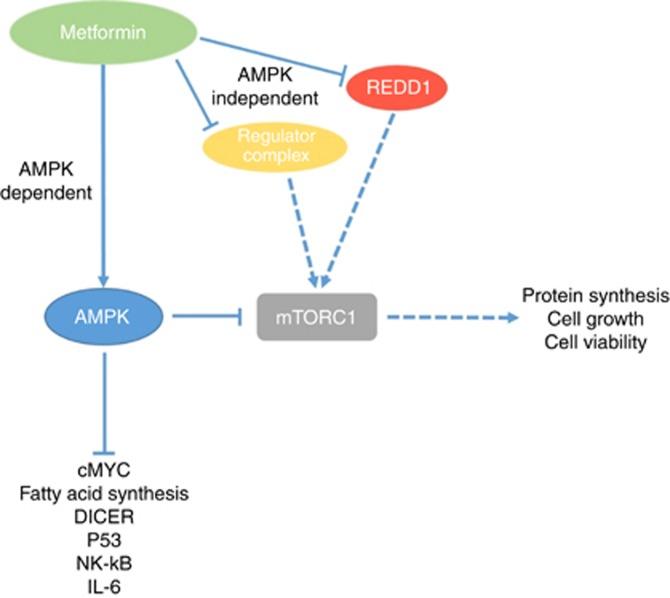
**The mechanism of action of metformin.** Metformin has both AMP-activated protein kinase (AMPK) dependent and independent mechanisms of action. Although, both these mechanisms can converge by inhibiting ‘mammalian target of rapamycin complex 1’ (mTORC1). AMPK is activated by metformin, resulting in downregulation mTORC1 and subsequent inhibition of tumour progression. Furthermore, metformin inhibits both the ragulator complex and ‘regulated in development and DNA damage response 1’ (REDD1) which subsequently results in inhibition of mTORC1.

Metformin is a biguanide that is typically used as an oral anti-hyperglycaemic in patients with type 2 diabetes mellitus. Metformin acts to lower plasma glucose levels by decreasing hepatic gluconeogenesis, decreasing intestinal glucose absorption and increasing peripheral glucose uptake (Lee and Kim, 2014). Epidemiological studies and subsequent meta-analyses, suggest that metformin reduces the risk of developing a number of malignancies such as breast, gastrointestinal, lung and hepatocellular carcinoma (Rizos and Elisaf, 2013). More recently, its role as adjunct to therapy for the treatment of several cancers has been explored (Coyle *et al*, 2016). In colorectal cancer, metformin’s anti-neoplastic effects are thought to be derived through a number of mechanisms. Primarily metformin can directly or indirectly activate 5' adenosine monophosphate-activated protein kinase (AMPK), which leads to downregulation of mammalian Target Of Rapamycin complex 1 (mTORC1) and resultant inhibition of tumour progression or induction of apoptosis (a review of the mTOR pathway (Guertin and Sabatini, 2007); Figure 4). Furthermore, AMPK independent regulation of mTORC1 by metformin occurs through REDD1 (regulated in development and DNA damage responses 1) or by Rag proteins in the ‘Ragulator complex’ (Pierotti *et al*, 2013).

Functional studies in colorectal cancer models have shown that metformin can potentiate the effects of other anti-cancer therapies such as mesalazine (Saber *et al*, 2016). Alongside this, metformin has been shown to induce radiosensitivity *in vitro*, in a radioresistant p53 colorectal cancer cell line, by prolonging cell-cycle arrest and inhibiting DNA repair proteins (Jeong *et al*, 2015). Radiosensitisation has also been reported to occur by metformin through decreasing cancer oxygen consumption thereby lessening the tumour promoting effects of tumour hypoxia (Zannella *et al*, 2013). In addition, this work was corroborated in 3D tumour spheroids which showed improved responses to radiation in colorectal cancer cell lines after metformin treatment had reduced spheroid oxygen consumption rates (Ashton *et al*, 2016). These *in vitro* reports highlight a novel role for metformin not only as a cancer risk-reducing drug but also one that could be used as an adjunct to conventional neo-adjuvant or adjuvant therapies in rectal cancer.

A clinical study by Skinner *et al* examined the effect of metformin use on response to neoadjuvant therapy (Skinner *et al*, 2013). Patients with rectal adenocarcinoma were divided into 3 groups: non-diabetics (422 patients), diabetics not taking metformin (40 patients) and diabetics on metformin (20 patients). All patients had neoadjuvant chemoradiation, with radiotherapy administered with 3D conformational technique and chemotherapy given with either 5FU (53%) or capecitabine (45%). Across all groups, there was a 17% rate of pCR. Diabetics on metformin had a higher rate of pCR than diabetics not on metformin and the non-diabetic patients, (35 *vs* 7.5 *vs* 16.6%, *P*=0.03). ANOVA confirmed that being diabetic and taking metformin was significantly associated with pCR (*P*=0.03). Metformin use was associated with a higher overall (*P*<0.001) and disease-free (*P*<0.001) survival, at both five and ten years, compared with diabetics not taking metformin. Interestingly, diabetic patients not taking metformin had a greater median tumour size (6 cm) than those in the diabetic on metformin (4.5 cm) and non-diabetic (5 cm) groups (*P*=0.01) possibly leading to confounding. Crucially there was no control experimental arm analysing response in patients given metformin who were not diabetic, something only achievable as part of a randomised control trial.

Oh *et al* (2016) analysed the role of metformin as an adjunct to radiation, using similar patient groups as above (Skinner *et al*, 2013). The crossover design resulted in two cohorts of patients, in which there were no significant differences in tumour characteristics, although the non-diabetic group was younger (*P*<0.001) and had a lower BMI (*P*=0.012). All patients had T3 or T4 tumours, were node positive, and had tumours <12 cm from the anal verge. They received radiotherapy (44–54 Gy fractionated as 1.2–2 Gy for 5 days a week) and chemotherapy (either 5FU and leucovorin (66%) or capecitabine). Tumour response was graded as either a decrease in T-stage, a decrease in N-stage, or a pCR. The study also assessed the pathological stage of response according to Dworak’s tumour regression grade (TRG) (Table 3) (Dworak *et al*, 1997). For comparative analysis the TRG was categorised as ‘non responders’ (TRG 0–2) and ‘responders’ (TRG 3–4).

On multivariate analysis, in both time cohorts, there was a significantly higher rate of TRG 3–4 (good/complete response) in the metformin group, (*P*=0.019 and *P*=0.044), although no difference in pCR. In the later cohort, metformin use was predictive of T-stage downstaging (*P*=0.013), but there was no significant difference in N (nodal status) downstaging. For the earlier cohort the opposite was true; metformin use was associated with N-downstaging (*P*=0.003) but not significant T-stage downstaging.

There was no significant difference in local recurrence-free survival, disease-free survival or overall survival between the groups. Metformin use was not a significant predictor for any type of survival in a Cox proportional hazards model.

### Further adjuncts to neoadjuvant therapies

Three papers were identified that outlined preliminary investigations (phase I or II studies) piloting non-chemotherapeutic agents as adjuncts to neoadjuvant chemo-radiotherapy (Wang *et al*, 2014; Illum *et al*, 2015; Stoffregen *et al*, 2016). Illum *et al* conducted a phase I, feasibility, cohort study investigating the safety, toxicity and optimum dose for nitroglycerin patches (nitric oxide donor) when used as an adjunct to neoadjuvant 5-fluorouracil and radiation in rectal cancer. There was no control group for comparison of outcomes, but the patches were well tolerated in the 13 patients studied, with minimal side effects and an overall pCR rate of 17%.

Supplementation with folic acid and vitamin B_12_ in patients receiving Permetrexed (a multitargeted antifolate drug) as a single neo-adjuvant chemotherapeutic agent (±radiotherapy) for rectal cancer, was assessed by Stoffregen *et al* (2016), They concluded that the regimen was feasible and tolerable. Levels of ‘reduced folates’ in tumours and various markers of vitamin metabolism were recorded and noted to fluctuate with the adjunct treatment, although this is of uncertain clinical value and is being further investigated.

Finally, further work examined the use of anti-inflammatory drugs; Wang *et al* conducted a phase II study examining the use of the NSAID celecoxib (400 mg per day) in patients receiving neoadjuvant treatment for locally advanced rectal cancer (Stage II or III). Of the 47 patients included (who received celecoxib, CRT and surgery), pCR rate was 13% (*n*=6) and tumour downstaging occurred in 81% (*n*=38). Tumour tissue was analysed for COX-2 expression; high levels of COX-2 following treatment were associated with reduced pelvic control and poorer disease-free and overall survival, than in those with low COX-2 expression. However, the use of celecoxib is limited given the significant increase in vascular and cardiac side effects (Bhala *et al*, 2013).

## Conclusion

Tumour regression following neo-adjuvant chemo-radiotherapy has been shown to be significantly associated with five-year overall survival, disease-free survival and local recurrence (Mace *et al*, 2015). There is, therefore, increasing interest in developing our understanding of treatment adjuncts to enhance tumour response to therapy. Current evidence suggests that the use of adjuncts to neoadjuvant treatment may improve outcomes by increasing the likelihood of pathologically complete or near-complete response in patients with rectal cancer.

If pCR truly allows a ‘watch and wait’ strategy, and if tumour downstaging facilitates local excision, such as TEMS/TEO, without affecting survival, then any treatment that may augment pCR rates should be fully explored.

Drug repurposing provides the opportunity to quickly translate basic science research into clinical therapies. Moreover, the repurposing of common drugs like aspirin, statins and metformin is promising, as the drug profile and associated side effects, risks and tolerance are well documented. However, the evidence for their routine use is as yet limited. All data we have analysed are within retrospective cohort studies in patients taking the drug for other indications.

The studies we have identified support the notion of a treatment effect of statins, aspirin and metformin. Each of these adjuncts appear to be associated with increased downstaging of tumours (supported in all publications), however the association with pCR is less clear. In only one report (Armstrong *et al*, 2015) statin use was identified on multivariate analysis as being an independent predictor of pCR.

Despite this potential, the literature contained only a small number of relevant studies, assessing three main adjuncts and used a variety of outcome measures; thus the heterogeneity of results meant that it was not possible to perform a meta-analysis on the data. Furthermore, any new drugs given to patients already burdened by a diagnosis of cancer, radiotherapy treatment and the side effects of long course chemotherapy need to have proven efficacy. There is a clear need for prospective randomised controlled trials to investigate these adjuncts, singularly or in combination, to determine whether they confer and increase in response to neo-adjuvant therapy or a long-term effect on survival.

## Figures and Tables

**Table 1:** Risk factors associated with local recurrence following rectal cancer surgery as predicted following staging using MRI pelvis, adapted from NICE guidelines 2014

**Characteristics of rectal tumours predicted by MRI**		
**Low**	**Moderate**	**High**
cT1-3a No lymph node involvement	cT3b or greater Surgical margin not threatened Any suspicious lymph node NOT threatening surgical margin Extramural vascular invasion	Threatened (<1 mm) or breached resection margin Low tumours enchroaching inter-sphincteric plane or levator involvement

Abbreviation: MRI=magnetic resonance imaging.

The risk of local recurrence helps to determine the use of neo-adjuvant therapy in rectal cancer.

**Table 2:** The American Joint Committee on Cancer (AJCC) criteria for determining pathological response to neo-adjuvant radiotherapy in patients with rectal cancer, as determined by histological analysis after tumour resection, (Mace *et al*, 2015)

**AJCC criteria for pathological response to neo-adjuvant radiotherapy**			
**0**	**1**	**2**	**3**
No viable tumour cells remaining	Single or small groups of tumour cells	Residual cancer outgrown by fibrosis	Minimal or no treatment effect

Pathological complete response (pCR) equates to a score of 0.

**Table 3:** Dworak’s tumour regression grade

**Dworak’s tumour regression grade**				
**0**	**1**	**2**	**3**	**4**
No regression	Dominant tumour mass with obvious fibrosis and/or vasculopathy	Dominantly fibrotic changes with few tumour cells or groups (easy to find)	Very few (difficult to find microscopically) tumour cells in fibrotic tissue with or without mucous substance	No tumour cells, only fibrotic mass (total regression or response)

Grading of regression was determined histologically. Tumours were graded according to the difficulty or ease by which a pathologist was able to find tumour cells within the resected specimen. (Dworak *et al*, 1997).
